# From data to insights: how natural language processing and structured reporting advance data-driven radiology

**DOI:** 10.1007/s00330-023-10242-w

**Published:** 2023-10-02

**Authors:** Matthias A. Fink

**Affiliations:** 1 Clinic for Diagnostic and Interventional Radiology, University Hospital Heidelberg, Heidelberg, Germany; 2 Translational Lung Research Center Heidelberg, Member of the German Center for Lung Research, Heidelberg, Germany

The past year has seen the most visible advances in what is known as natural language processing (NLP), the branch of artificial intelligence (AI) that focuses on how machines can understand and generate language like humans do. The current hype around NLP in general is largely due to the recent emergence of large language models (LLMs), which now allow anyone without technical expertise to engage with the most advanced NLP algorithms using an easy-to-use browser-based interface. Over the last decade, the progress of NLP has been greatly accelerated by the increasing computing power of hardware and the associated development of deep learning techniques such as the transformer architecture used in the existing generative pretrained transformer (GPT)–based LLMs [1]. Given the rapid growth of digital data and the increasing need for automated language processing, NLP has become an indispensable technology in various industries—not only in healthcare, but also in finance, education and marketing.

However, it is important to note that LLMs, as the latest generation of language processing models, are only one pillar of an interdisciplinary research field dedicated to the development and application of NLP algorithms [2]. These include invaluable tools for processing, aggregating and simplifying text corpora, and depending on the underlying NLP problem, a variety of methods from a broad NLP toolbox can be selected to facilitate its application for the benefit of radiology. Radiologists who understand the potential and limitations of NLP will be better equipped to evaluate NLP models, understand how they can improve clinical workflow and facilitate research efforts involving large amounts of textual data.

Current NLP methods used for text processing and analysis in radiology range from traditional rule-based systems (e.g. string matching [3]) to feature-rich learners (e.g. conditional random fields, support vector machines [4]) and deep learning methods such as convolutional and transformer-based neural networks [5], including cutting-edge LLMs [6]. A well-established NLP technique is Word2Vec, developed by Google in 2013, which uses a two-layer neural network to learn word associations from a large corpus of text without additional user input [7]. Once trained, such a model can detect synonyms or suggest additional words for a partial sentence. The vectors produced by Word2Vec capture the semantic and syntactic qualities of words, allowing for the measurement of their semantic similarity.

In their study, Vosshenrich et al [8] used Doc2Vec, an NLP algorithm that generalises Word2Vec to encode whole documents rather than individual words. The resulting vector representations encapsulate various relationships between documents and may prove useful in tasks such as quantifying similarities or differences between text corpora. The authors conducted a retrospective analysis of a large cohort of nearly 750,000 institutional radiology reports over a 10-year period, which were processed by Doc2Vec and converted into multidimensional vectors. The dimensionality of the vectors was then reduced to 2D data using a non-linear dimensionality reduction method known as t-distributed stochastic neighbour embedding (t-SNE) to facilitate visualisation and statistical analysis between two different types of radiology reports: free-text and (semi)structured reports. Based on the analysed differences in the spread and centroids of the document vectors, the authors found that structured reports had higher linguistic similarity and better linguistic discrimination compared to free-text reports.

While these results may not be surprising given the underlying transition from prose text to consistent reporting organisation and terminology, they do provide quantifiable evidence that structured reporting can improve the standardisation and distinguishability of reporting language in radiology, which could also facilitate automated data postprocessing.

The choice of reporting style has major implications, as it conveys the essence of the radiologist’s interpretation of medical images and thus contributes key diagnostic information to the therapeutic decision-making process. The clarity and completeness of the radiology report, as the primary means of communication between clinicians and between clinicians and patients, can therefore have a significant impact on both the transmission of diagnostic information and the quality of report translation into machine-readable data. Transforming radiology data into a digital stream of structured diagnostic information and quantitative image data, supported by tailored NLP extraction methods, would facilitate integration with other diagnostic modalities and promote data-driven health care, integrated diagnostics and visual grounding approaches in radiology at scale. However, developing reliable NLP pipelines to retrieve key information and feed downstream processing, such as weakly supervised learning frameworks for AI model building, integration with aggregated clinical data or correlation with imaging biomarkers, will become easier as we structure radiology reporting and procedures during primary data collection. By moving towards structured reporting in clinical practice, and leveraging rapid advances in NLP technology, we will be able to unlock the full potential of data in our field. This will ultimately lead to system improvements for patients, clinicians and institutions, improving the quality of care.

## Declarations

### Guarantor

The scientific guarantor of this publication is Matthias A. Fink.

### Statistics and biometry

No complex statistical methods were necessary for this paper.

### Methodology

• Commentary
